# Recent advances in the management of pediatric acute lymphoblastic leukemia

**DOI:** 10.12688/f1000research.9548.1

**Published:** 2016-11-04

**Authors:** Jan Starý, Ondřej Hrušák

**Affiliations:** 1 Department of Pediatric Hematology and Oncology, 2nd Faculty of Medicine, Charles University and University Hospital Motol, Prague, Czech Republic

**Keywords:** paediatric, leukaemia, ALL, HSCT, immunotherapy, monoclonal antibodies

## Abstract

Acute lymphoblastic leukemia (ALL) is the most common malignancy in childhood. Despite enormous improvement of prognosis during the last half century, ALL remains a major cause of childhood cancer-related mortality. During the past decade, whole genomic methods have enhanced our knowledge of disease biology. Stratification of therapy according to early treatment response measured by minimal residual disease allows risk group assignment into different treatment arms, ranging from reduction to intensification of treatment. Progress has been achieved in academic clinical trials by optimization of combined chemotherapy, which continues to be the mainstay of contemporary treatment. The availability of suitable volunteer main histocompatibility antigen-matched unrelated donors has increased the rates of hematopoietic stem cell transplantation (HSCT) over the past two decades. Allogeneic HSCT has become an alternative treatment for selected, very-high-risk patients. However, intensive treatment burdens children with severe acute toxic effects that can cause permanent organ damage and even toxic death. Immunotherapeutic approaches have recently come to the forefront in ALL therapy. Monoclonal antibodies blinatumomab and inotuzumab ozogamicin as well as gene-modified T cells directed to specific target antigens have shown efficacy against resistant/relapsed leukemia in phase I/II studies. Integration of these newer modalities into combined regimens with chemotherapy may rescue a subset of children not curable by contemporary therapy. Another major challenge will be to incorporate less toxic regimens into the therapy of patients with low-risk disease who have a nearly 100% chance of being cured, and the ultimate goal is to improve their quality of life while maintaining a high cure rate.

## Introduction

Acute lymphoblastic leukemia (ALL) accounts for 25% of all childhood cancers and its outcome significantly influences the overall treatment results in pediatric oncology. The improvement of prognosis in childhood ALL is one of the most successful stories of modern medicine. An almost uniformly fatal disease at the beginning of the 1960s has transformed into a curable disorder in more than 90% of children with contemporary therapy. The major reason for improved survival is a decreased risk of relapse ^1, 2^. The outcome continues to improve at the beginning of the 21st century. Moreover, this progress takes place not only in high-income countries but also in countries with limited resources because conventional chemotherapy is inexpensive and readily available worldwide ^3^.

## Early response to treatment

Since the beginning of the 2000s, patients have been stratified into risk groups according to the early treatment response evaluated by minimal residual disease (MRD). It has been determined by detection of aberrant leukemic phenotype by flow cytometry or by specific rearrangements of immunoreceptor genes *IgH*/ *TCR* (heavy immunoglobulin/T-cell receptor) ^4^. MRD has been confirmed in multivariate analysis as the most significant prognostic factor within all immunophenotypes and genetic subgroups. MRD is first monitored during and at the end of induction ^5, 6^. Patients with a slow early response are stratified into the high-risk group. They profit from a more intensive post-induction treatment and augmented delayed intensification ^7– 10^. Rapid early responders to combined chemotherapy with B-cell precursor (BCP)-ALL benefit from early intensification of post-induction therapy, not from a prolonged double-delayed (augmented) intensification ^11, 12^. MRD performed about 3 months from diagnosis—that is, after early post-induction treatment—identifies a subset of patients with inferior outcome on Berlin-Frankfurt-Münster (BFM) protocols ^13, 14^. Treatment of T-cell (T)-ALL stratified according to treatment response has led to the excellent event-free survival (EFS) and overall survival (OS) of 85% and over 90%, respectively ^15^, although T-cell immunophenotype remains an adverse risk factor in multivariate analyses of clinical trials ^1, 14^. Stratification according to MRD response helps to improve prognosis of T-ALL patients in general but also in subsets, such as in the recently identified early T-cell precursor (ETP)-ALL. ETP-ALL patients tend to respond more slowly to induction therapy, and initial studies reported poor outcome ^16^. When stratified according to MRD response, their outcome is comparable to those with non-ETP-ALL ^15, 17^.

## Contemporary therapy

Optimization of standard chemotherapy in academic randomized clinical studies improves the outcome even at the beginning of the 21st century. Substituting dexamethasone for prednisone for 3 weeks in the context of 2 years of BFM multi-agent therapy reduced the relapse risk by one-third ^18^. On the other hand, dexamethasone also accounts for both long- and short-term side effects, many of which are dependent on dose intensity ^19^. The immunosuppressive action of dexamethasone, alongside the use of anthracyclines, contributed to higher incidence of infection-related mortality compared with prednisone and thus resulted in no difference in OS between both arms. High-dose methotrexate 5 g/m ^2^ (HD MTX) in consolidation reduces bone marrow (BM) and extramedullary relapses more efficiently than lower escalating doses of intravenous MTX without leucovorin rescue (followed by l-asparaginase, Capizzi regimen) in BCP-ALL ^20^. l-asparaginase has been a key component of all treatment regimens for childhood ALL since the 1960s. Its optimal dose, preparation, and route of administration remain uncertain ^21^. Prolonged and intensified therapy with l-asparaginase improves the outcome of children with ALL. Administration of l-asparaginase can be hampered by allergic reactions. Clinical allergy is associated with inactivation of l-asparaginase by antibodies. Apart from overt allergy, antibodies can also cause a so-called silent inactivation, in which l-asparaginase is neutralized subclinically. Monitoring of l-asparaginase blood levels is useful because it detects silent inactivation ^22^. Pegylated *Escherichia coli* l-asparaginase ( l-asparaginase coupled to polyethylene glycol) has a longer half-life and is potentially less immunogenic than native *E. coli* l-asparaginase and has been used with increasing frequency in frontline pediatric treatment regimens ^23^. Following allergy to pegylated l-asparaginase, Erwinia l-asparaginase can be substituted and achieves therapeutic activity ^24^.

Cranial irradiation is associated with a higher incidence of secondary malignancies and cognitive impairment in young children ^25, 26^. The use of dexamethasone, HD MTX, and intrathecal triple therapy in central nervous system (CNS) leukemia treatment has enabled reduction or even omission of cranial irradiation without compromising OS ^27, 28^. In a recently published meta-analysis, cranial radiotherapy (CRT) was associated with reduced risk of relapse only in patients with overt CNS leukemia at diagnosis ^29^. Omission of CRT from childhood ALL therapy results in improved cognitive outcome. However, despite this improvement, survivors continue to demonstrate the elevated risk for attention deficits when compared with a reference population of children ^30^.

The indication of hematopoietic stem cell transplantation (HSCT) in first complete remission (CR) is recommended for a subgroup of children (approximately 5%) who are resistant to current chemotherapy (induction failure, persistent high MRD) ^31, 32^.

Maintenance treatment with mercaptopurine and MTX is an integral part of ALL treatment. Lower adherence to oral mercaptopurine increases relapse risk ^33^. The addition of vincristine plus steroid pulses in maintenance treatment still may be useful in cases where less intensive early therapy is used ^34^. Children with intermediate-risk ALL who received intensive chemotherapy on the basis of BFM protocols did not benefit from intensification of the maintenance therapy with pulses of vincristine and dexamethasone ^35^. Some subgroups of patients with unfavorable biology, like deletion of the gene for transcription factor *IKZF1*, might benefit from pulses during maintenance even on treatment with BFM backbone ^36^.

## Treatment-related mortality

The incidence of treatment-related mortality (TRM) on contemporary ALL trials is reported to be between 2 and 4%. The most common cause of death is infection. The 5-year EFS of children with low-risk ALL who have favorable cytogenetics and good response to treatment is higher than 90%, and death from TRM is as common as relapse. As such, reducing infection-related mortality is a paramount concern when improving outcomes in childhood ALL. Patients at highest risk of infection-related TRM are children older than 10 years, slow early responders, and those with Down syndrome (DS) ^37, 38^. Lower socioeconomic status, non-compliance to therapy, and (in countries with limited resources) malnutrition all increase the risk of treatment failure ^39, 40^.

## Biologic and genetic features: targeted therapy

Good risk prognostic genetic biomarkers of BCP-ALL are t(12;21) (ETV6-RUNX1) and high hyperdiploidy (51–65 chromosomes) ^41^. Patients with either of these abnormalities have a 5-year OS of over 90%. Five chromosomal abnormalities—MLL (KMT2A) translocations, t(9;22) (BCR-ABL1), t(17;19) (TCF3-HLF), near haploidy (fewer than 30 chromosomes), and low hypodiploidy (30–39 chromosomes)—are well-recognized prognostic biomarkers of high-risk disease at all ages ^42^. Previously reported uniformly poor outcome of patients with hypodiploidy of less than 45 chromosomes might be improved by MRD-stratified treatment ^43^. Approximately 30% of pediatric patients with BCP-ALL are screened negative for established diagnostic and prognostic markers. They are referred to as B-other ALL. Approximately 50% of B-other cases have a gene expression profile similar to that of BCR-ABL1-positive ALL. These cases form a subgroup that has been termed BCR-ABL1-like or Ph-like ^44, 45^. They frequently harbor alterations of *IKZF1* or other B-lymphoid transcription factor genes. Stratification of these patients according to MRD helps to improve previously reported poor outcome of this subgroup ^46^. Kinase-activating gene fusions detected in this subgroup are collectively found in 1 to 2% of BCP-ALL cases and can be targeted with tyrosine kinase inhibitors (TKIs) ^47^. Patients failing initial treatment might profit from the introduction of TKIs to high-risk chemotherapy. Up to 5% of childhood ALL cases have JAK-STAT signaling pathway-activating fusions ( *CRLF2*, *JAK2*, and *EPOR*), which appear to be targetable by JAK inhibitors ^48^.

## Philadelphia chromosome-positive acute lymphoblastic leukemia

HSCT has been the gold standard treatment for maintaining CR in Philadelphia chromosome-positive (Ph ^+^) ALL since the 1990s; however, its importance may change in the era of TKIs. The onset of targeted therapy exemplified by TKIs has revolutionized the therapy of Ph ^+^ ALL. TKIs are introduced early in induction and continue through maintenance treatment. Length of treatment and cumulative dose of TKIs seems to play a role in the achievement of a better outcome ^49, 50^. MRD is considered to be essential for risk group stratification. Discrepancies in MRD using *IgH/TCR* and BCR-ABL1 fusion indicate that lineages other than BCP may also be affected by the disease ^51^. The best way to incorporate TKIs, conventional chemotherapy, and HSCT is still unclear. With current approaches, which are developed from high-risk protocols, acute toxicity is a significant problem. Future trials will examine the necessary chemotherapy dose intensity alongside TKI therapy and address in which subgroups HSCT is needed; with regard to TKIs, which agent to use, how long application should be, and what the role of post-HSCT is must be further investigated ^52^.

## Clinical features: age and Down syndrome

Most of the adolescents older than 15 years can be cured with risk-adjusted intensive therapy without HSCT. They show higher rates of severe infections, osteonecrosis, thrombosis, and hyperglycemia ^53, 54^. On the other hand, the outcome of infants younger than 1 year old is not improving. MLL gene rearrangement, which is associated with chemoresistance to conventional chemotherapy, is present in 80% of them. Their leukemic cells display a global hypermethylated genomic state. It explains remarkable sensitivity to demethylating agents ^55^. Their potential to improve the outcome of the infant ALL cases will be verified in clinical trials.

Children with DS-ALL have a higher cumulative incidence of relapses and therapy-related mortality (infectious deaths) than non-DS patients. They have low incidence of prognostically advantageous genetic subtypes (ETV6-RUNX1 fusion, hyperdiploidy 51–65 chromosomes). Higher risk of toxic complications puts pressure on physicians to reduce the treatment intensity in DS-ALL. However, even in DS, unmodified risk-adjusted ALL treatment, combined with good supportive care, is indicated ^56^.

## Leukemias of ambiguous lineage

Up to 5% of acute leukemia cases present with some kind of lineage ambiguity. Among the four subsets of ambiguous lineage leukemias, the most prevalent are those classified by the presence of too many phenotype features of the opposite lineage (ALL versus acute myeloid leukemia). The existing definitions are arbitrary and sometimes are criticized for including too few markers (the World Health Organization definition, now most widely used) or too many markers (the previous EGIL—European Group for the Immunological Classification of Leukemias—definition) ^57, 58^. Irrespective of the definition, the prognosis of children with this type of ambiguity was slightly poorer when compared with other cases receiving the same treatment ^59, 60^. Much rarer subtypes are bilineal (biclonal) leukemias, in which two or more clones belonging to different lineages coexist at diagnosis. Interestingly, emerging studies show that despite phenotypic differences, the two clones often share genetic features ( 61 and Thomas Alexander, personal communication). A third subtype, also uncommon, is the undifferentiated leukemia. Patients with this subset do not fulfill the existing criteria for ALL or for acute myeloid leukemia. In such situations, infiltration of hematopoietic organs by another malignancy should always be considered. The fourth subtype is represented by leukemias that change their phenotype from one lineage to another during the beginning of treatment. Historically, this feature has mostly been referred to as being associated with MLL rearrangements or BCR/ABL1 ^62^. More recently, lineage switching toward monocytic lineage has been described mostly in ALLs with aberrant CD2 at presentation ^63^. This phenomenon is present in varying degrees in up to 4% of BCP-ALL cases and is mostly temporary, not requiring major changes of treatment; however, monocytosis as well as monocytoid organ infiltration may be clinically significant, and development into monocytic leukemia has been observed in one case ^63^. Besides CD2, such “swALL” cases are associated with changes in CEBPA methylation ^63^ and overlap with ERG-deleted ALLs ^64^. Overall, owing to their rarity, ambiguous lineage leukemias should be studied cooperatively. An international study currently taking place addresses the treatment outcome of over 270 cases and their molecular background and aims to find the causal factors as well as optimal treatments ^65^.

## Relapsed acute lymphoblastic leukemia

Relapse is diagnosed in fewer than 15% of children. Relapsed disease tends to be more drug resistant than is newly diagnosed ALL, as shown by lower disease clearance and reduced CR rate, especially in early relapses ^66, 67^. When diagnosed, most ALL cases are oligoclonal. A patient’s sensitivity or resistance to therapy is affected by genetic variations in individual subclones, and these can influence a subsequent clonal evolution during treatment as well ^68^. Major diagnostic clone at diagnosis is eradicated by chemotherapy in the majority of cases. A substantial part of relapsed ALL cases arise from minor subclones already present at diagnosis. Some abnormalities gained under chemotherapy pressure confer resistance to important components of combined chemotherapy, like nucleoside analogues ( *NT5C2*, *PRPS1* mutations) and glucocorticoids ( *CREBBP* mutation) ^69– 72^. Despite this, the optimization of combined chemotherapy might improve the outcome. The UK ALL R3 randomized trial for patients in first relapse compared two anthracyclines, mitoxantrone and idarubicin, in induction. Relapse incidence was significantly lower in the group treated with mitoxantrone and translated into a clear survival advantage of more than 20%, one of the largest improvements ever achieved by a single drug. Surprisingly, no difference in MRD could be detected between two randomized groups. Three-year OS was 69% in the mitoxantrone group ^73^. The most frequent type of relapse is late BM relapse, more than 6 months from completion of treatment in BCP-ALL. Contemporary chemotherapy combined with HSCT in MRD poor responders to induction treatment is able to cure 70% of children ^74^. In this group, deletion of *IKZF1* transcription factor and alteration of *TP53* identify patients with significantly inferior outcome ^75^. The outcome of early BM relapses of BCP-ALL (during treatment or less than 6 months from the completion of treatment) and all BM relapses of T-ALL is worse. HSCT is indicated in all of these patients achieving remission. Only one-third of them are cured ^76^. Deletion of *IKZF1* is strongly predictive of a second relapse after HSCT ^75^. *IKZF1* and *TP53* represent relevant prognostic factors that should be considered in the assessment of children with relapsed ALL. The outcome of isolated and combined extramedullary relapses depends on the interval between diagnosis and relapse. HSCT may improve the outcome of those with highest risk.

The results of allogeneic HSCT are steadily improving. There is no difference in outcome between HSCT from matched sibling donors (MSDs) and unrelated main histocompatibility antigen-identical 9–10/10 volunteers (matched unrelated donor [MUD]). Transplant-related mortality is now below 10% in MSD and 20% in MUD HSCT. The likelihood of being cured by HSCT performed in second remission is about 60% ^31^. Standard conditioning regimens include total body irradiation, which is associated with significant risk of secondary cancer. Substituting chemotherapy for total body irradiation is currently being evaluated by a randomized study.

## Immunotherapy

The intensity of current front-line therapy has reached its threshold. Further intensification is not realistic. The majority of children with early BM relapse currently have little chance to be cured. There is growing evidence that targeted treatment and immunotherapy have the potential to improve the outcome of childhood ALL with reduced toxicity. A novel method of cellular therapy is based on the use of adoptively transferred T lymphocytes which were modified *in vitro* prior to transfer to express an artificial signaling molecule designated chimeric antigen receptor (CAR). The CAR redirects the specificity of the modified T lymphocytes to surface antigens expressed by malignant cells. The most successful example of CAR-based immunotherapy is the treatment of BCP-ALL by targeting of the surface antigen CD19. In a pilot study, CR was achieved in 90% of 30 children and adults with highly refractory disease ^77^. CAR cells can cross the blood-brain barrier and eliminate leukemic cells in the cerebrospinal fluid. CAR cells of the third generation are able to engraft and persist for at least several months ^78^. The most common acute toxicity of CAR T cells is cytokine release syndrome. A wide variety of cytokines, including interleukin-6, interferon-gamma, and tumor necrosis factor, are elevated in the serum of patients experiencing fever, tachycardia, hypotension, and other toxicities. Higher disease burden predicts more toxicity. The interleukin-6 receptor antagonist tocilizumab is widely used for toxicity following CAR infusion ^79^. Randomized studies are needed to demonstrate whether they will be able to substitute HSCT.

Blinatumomab, a bi-specific anti-CD19/CD3 chimeric antibody, draws malignant B cells in close proximity to CD3-positive T cells. Activated T cells effectively induce serial target cell killing. In current clinical trials, blinatumomab is administered over a 28-day continuous infusion, followed by a 14-day rest period. In a phase I/II study of blinatumomab in children, objective responses were noted in 43% patients, including negative MRD in 39% of patients. Optimal placement of immunotherapy with blinatumomab or other conventional immunomodulating approaches or both has yet to be determined ^80, 81^.

The anti-CD22 immunoconjugate inotuzumab ozogamicin is an antibody-drug conjugate, consisting of humanized anti-CD22 monoclonal antibody joined to the cytotoxic agent calicheamicin by a non-immunogenic linker. After inotuzumab calicheamicin binds to CD22, rapid internalization of the conjugated calicheamicin occurs, targeting tubulin and inducing DNA breakage, apoptosis, and cell death. The response rate in adults with refractory/relapsed ALL is 80%, and clinical studies in children were launched recently ^82^.

## Conclusions

Despite advances in targeted therapy approaches, conventional chemotherapy is unlikely to be replaced and this is because of its impressive cure rate in ALL. The task in coming years will be to compare immunotherapy with standard chemotherapy on a randomized basis in frontline treatment and in the treatment of first relapse. The goals are to also cure those children in whom combined chemotherapy fails and to reduce the toxicity that is responsible for reducing the quality of life for those who are cured.

## Abbreviations

ALL, acute lymphoblastic leukemia; BCP, B-cell precursor; BCP-ALL, B-cell precursor acute lymphoblastic leukemia; BFM, Berlin-Frankfurt-Münster (study group); BM, bone marrow; CAR, chimeric antigen receptor; CNS, central nervous system; CR, complete remission; CRT, cranial radiotherapy; DS, Down syndrome; DS-ALL; acute lymphoblastic leukemia in children with Down syndrome; EFS; event-free survival; ETP, early T-cell precursor; ETP-ALL, early T-cell precursor acute lymphoblastic leukemia; HD, high-dose; HSCT, hematopoietic stem cell transplantation; IgH/TCR, heavy immunoglobulin/T-cell receptor; MRD, minimal residual disease; MSD, matched sibling donor; MTX, methotrexate; MUD, matched unrelated donor; OS, overall survival; Ph ^+^ ALL, Philadelphia chromosome-positive acute lymphoblastic leukemia; T-ALL, T-cell acute lymphoblastic leukemia; TKI, tyrosine kinase inhibitor; TRM, treatment-related mortality.
